# Gamma sensory entrainment for cognitive improvement in neurodegenerative diseases: opportunities and challenges ahead

**DOI:** 10.3389/fnint.2023.1146687

**Published:** 2023-04-17

**Authors:** Prangya Parimita Sahu, Philip Tseng

**Affiliations:** ^1^Graduate Institute of Mind, Brain and Consciousness, Taipei Medical University, Taipei, Taiwan; ^2^Brain and Consciousness Research Center, Shuang Ho Hospital, Taipei Medical University, Taipei, Taiwan; ^3^Cross College Elite Program, National Cheng Kung University, Tainan, Taiwan; ^4^Research Center for Mind, Brain and Learning, National Chengchi University, Taipei, Taiwan

**Keywords:** sensory entrainment, brain stimulation, 40 Hz stimulation, GENUS, visuoauditory stimulation

## Abstract

Neural oscillations have been categorized into various frequency bands that are mechanistically associated with different cognitive functions. Specifically, the gamma band frequency is widely implicated to be involved in a wide range of cognitive processes. As such, decreased gamma oscillation has been associated with cognitive declines in neurological diseases, such as memory dysfunction in Alzheimer’s disease (AD). Recently, studies have attempted to artificially induce gamma oscillations by using 40 Hz sensory entrainment stimulation. These studies reported attenuation of amyloid load, hyper-phosphorylation of tau protein, and improvement in overall cognition in both AD patients and mouse models. In this review, we discuss the advancements in the use of sensory stimulation in animal models of AD and as a therapeutic strategy in AD patients. We also discuss future opportunities, as well as challenges, for using such strategies in other neurodegenerative and neuropsychiatric diseases.

## Introduction

Synchronization of neural network oscillations within the gamma-band frequency range has been linked to critical bodily and cognitive processes. Because the functionality of gamma oscillations differs across brain regions, gamma oscillations has been extensively investigated in various brain regions, including the cortex, hippocampus, amygdala, striatum, and brainstem. These oscillations are broadly connected to sensation, recognition, memory, emotion, locomotion, and the control of sleep and wakefulness (Guan et al., 2022). Mechanistically, gamma oscillations have been proposed to mediate various cognitive processes through the synchronization of neural networks, which can affect the balance of neuronal excitation and inhibition (E/I). Pathologically, the loss of neurons in neurodegenerative diseases such as Alzheimer’s disease (AD) can also result in impaired E/I homeostasis and altered levels of gamma power (Ribary et al., 1991; Stam et al., 2002; Osipova et al., 2006; Rossini et al., 2006; van Deursen et al., 2008, 2011; Lewis et al., 2012; Başar et al., 2016; Başar et al., 2017; Mably and Colgin, 2018; Guan et al., 2022), eventually leading to the loss of structural and functional neuronal integrity (Querfurth and LaFerla, 2010).

## 40 Hz sensory stimulation

Recently, studies have explored the use of sensory stimulation at 40 Hz to boost gamma oscillations in the brain through neural entrainment. Neural entrainment can be broadly defined as the progressive synchronization of neural network rhythms, while sensory entrainment refers to when these rhythms correspond to external stimuli. Sensory entrainment can occur through any sensory organ, though in neuroscience research it is typically achieved *via* auditory and visual stimuli in the forms of sound and light flickers, respectively, at the desired frequency (Sameiro-Barbosa and Geiser, 2016; Hanslmayr et al., 2019; Lakatos et al., 2019). It is hypothesized that sensory stimulation can modulate the functional connectivity between pyramidal neurons and inhibitory interneurons, such as parvalbumin, somatostatin, and vasoactive intestinal peptide interneurons, to regulate real-time interactions within neural circuits and maintain gamma band power.

One specific *de novo* strategy of sensory entrainment, called Gamma Entrainment Using Sensory Stimuli (GENUS), comprises both auditory and visual entrainment, and has been used in many studies on animal models of Alzheimer’s disease (AD) (Iaccarino et al., 2016; Adaikkan et al., 2019; Martorell et al., 2019; Chan et al., 2021a) as well as in AD patients (Suk et al., 2020; Chan et al., 2021b; He et al., 2021). The visual aspect of GENUS is delivered by placing the animals in a dark chamber and illuminating it with a light-emitting diode (LED) bulb that flickers at the desired frequency (e.g., 40 Hz). Similarly, the auditory aspect is delivered when the animals are placed in a dimly lit chamber insulated with soundproof foam and then subjected to tones of the desired frequency *via* speakers placed out of reach of the animals (Adaikkan et al., 2019; Martorell et al., 2019). It is important to note that 40 Hz is not referring to pitch; rather, a tone of any pitch can be played intermittently 40 times per-second. In humans, this visual auditory setup can be done with a portable head-mounted display (HMD) and earphones.

One of the initial and major studies on sensory stimulation was conducted by Iaccarino et al. (2016), who used a 5XFAD AD mouse model and manipulated the animal’s gamma oscillations through light flickers. The authors compared a range of gamma frequency entrainment, such as 20, 40, and 80 Hz, as well as random flickers and a dark (control) condition. They observed that only 40 Hz entrainment exhibited positive outcomes in the animal model, and not the other frequencies or the random frequency control. It is important to note that the 5XFAD mice harbor three FAD mutations in human APP, such as the Swedish (K670N and M671L), Florida (I716V), and London (V7171) mutations, and two FAD mutations in human PSEN1, such as M146L and L286V (Oakley et al., 2006). Therefore, this model displays significant beta-amyloid plaque formation and related pathophysiology and symptoms. Iaccarino and colleagues reported that only 1 h of 40 Hz visual entrainment stimulation could significantly reduce beta-amyloid plaque load in the visual cortex of the 5XFAD animal compared to dark controls. Mechanistically, this reduction was attributed to the activation and morphological transformation of microglia cells, increasing their phagocytic activities, as well as their increased interaction with beta-amyloid clusters. Furthermore, the application of 40 Hz entrainment to the same animals with a regimen of 1 h daily for 7 days further reduced the beta-amyloid load. This reduction included both soluble and insoluble Aβ1-40 and Aβ1-42 isoforms, leading to a decrease in AD pathophysiology and enhanced cognitive performance in the animals. The authors went ahead to show that the therapeutic effects of 40 Hz sensory entrainment were not limited to β-amyloid, but also could significantly reduce tau-phosphorylation in the visual cortex of a P301S tauopathy animal model, presumably also *via* activation of microglia with increased phagocytic activity (Iaccarino et al., 2016).

In another study (Adaikkan et al., 2019), the same group investigated the P301S tauopathy model and another model called CK-p25, which are known to exhibit neurodegeneration (synaptic and neuronal loss) and cognitive deficit at an early age due to tau hyperphosphorylation and formation of neurofibrillary tangles. The authors applied 40 Hz visual entrainment to these neurodegeneration animal models of AD and observed gamma entrainment at the range of 40 Hz in not only the visual cortex but also the hippocampus, prefrontal cortex, and somatosensory cortices. Such entrainments were also observed between these brain regions in addition to just the local areas, suggesting that 40 Hz gamma stimulation might increase functional neuronal connectivity between the brain areas—a hallmark of healthy cognitive functioning. When the stimulation was prolonged at a regimen of 1 h daily for 22 days to P301S animals and 6 weeks to CK-p25 animals, the authors observed significant reduction of neurodegeneration and related cognitive symptoms compared to no-stimulation animals. Importantly, such neuro-protective effects were not observed in 80 Hz stimulation, suggesting that the effect is specific to 40 Hz.

In one recent study by Martorell et al. (2019), the authors applied a series of auditory stimulations (8 Hz, 40 Hz, 80 Hz, Random stimulation, and No stimulation) 1 h daily for 7 days in 5XFAD AD mouse models, out of which only 40 Hz auditory stimulation showed significant reduction of β-amyloid plaques in comparison to other included stimulations, which is similar to the Iaccarino et al. (2016) study. Unlike Iaccarino et al.’s findings where visual stimulation only entrained the occipital cortex, Martorell et al. (2019) found that auditory stimulation not only entrained the auditory cortex, but also extended to the CA1 area of the hippocampus as well as the medial prefrontal cortex (mPFC). Consistently, the authors observed significant reduction of β-amyloid load in all aforementioned brain regions of 5XFAD mice caused by 40 Hz auditory GENUS application in comparison to random stimulation or no stimulation. The authors further confirmed that the reduced β-amyloid load in these areas was due to significantly increased glial cell response caused by 40 Hz GENUS. In addition, the areas also exhibited enhanced vasculature and increased diameter, which further increased the association between β-amyloid plaques and the vasculature. The authors also applied 40 Hz GENUS to P301S tauopathy mice, which showed reduced tau-phosphorylation in the auditory cortex as well as the CA1 area of the hippocampus and mPFC. Performing cognitive tasks such as novel object recognition (NOR) and novel object location (NOL) in 5XFAD mice confirmed improved recognition and spatial memory in the animals treated by 40 Hz GENUS.

Importantly, Martorell et al. (2019) also used a combination of auditory and visual 40 Hz stimulations to 5XFAD mice 1 h daily for 7 days in a row, and observed significant reduction in β-amyloid load not only in sensory cortices but also in the whole neocortex. The reduction of Aβ plaques with such combined stimulation was significantly higher when compared to either auditory or visual stimulation alone. The authors conclude that the observed reduction of β-amyloid load in the entire neocortex was due to the clustering of several microglia cells around the Aβ plaques that strongly up-surged microglia response. Therefore, 40 Hz sensory entrainment in various animal models of AD has resulted in marked reduction of Aβ plaques and tau-phosphorylation due to increased microglia response and enhanced vasculature, as well as vasodilation, all of which ultimately resulted in improved cognition in animal models (Chan et al., 2021a). Other studies have also reported that such entrainment effect could be increased when combined with other factors such as exercise. A study used a 3x-TG murine AD model and applied 40 Hz light flickering for 3 months while the animals were subjected to daily physical exercise. The study reported that not only exercise or 40 Hz flickering could decrease Aβ plaques and tau-phosphorylation and lead to improved cognitive impairments such as spatial learning and long-term memory, the combination of 40 Hz flicker and exercise further enhanced the efficacy (Park et al., 2020).

In an attempt to replicate the study by Iaccarino et al. (2016), Bobola et al. (2020) used transcranial focused ultrasound (tFUS) pulsed at 40 Hz (400 μs pulses 5 s on and 5 s off) on the same 5xFAD mouse model for 1 h per day for 5 days. The results showed significant activation of microglia and their co-localization with Aβ plaques throughout the ultrasound-exposed brain (Bobola et al., 2020). Similarly, (Etter et al., 2019) used J20-APP animal models that display loss of spatial memory as well as decreased lower gamma amplitude and conducted optogenetic methods to activate medial septal parvalbumin (PV) interneurons in the hippocampus with different frequencies. The authors reported that gamma stimulation at only 40 Hz could rescue hippocampal slow gamma oscillation, thus enhancing spatial memory performance (Etter et al., 2019). In contrast, (Wilson et al., 2020) used 5xFAD mouse models and optogenetically activated PV + interneurons of the basal forebrain. Surprisingly, contrary to previous studies, the authors reported an increase in Aβ plaque deposition in the PV + neurons of the basal forebrain (Wilson et al., 2020). These seemingly contradictory results suggest that the exact mechanisms by which gamma oscillation is involved in AD pathology are still unclear and therefore require further studies. Furthermore, in another study using a two-vessel occlusion (2VO) cerebral ischemia mouse model, (Zheng et al., 2020) reported that visual stimulation with 40 Hz light flickering rescued the reduced gamma band power in the CA1 area of the hippocampus, thereby protecting the neurons from 2VO-induced degeneration. The mechanism of this protective attribute was found to be the 40 Hz sensory entrainment, which could augment RGS12-regulated CA3-CA1 pre-synaptic N-type calcium channel-dependent short-term synaptic plasticity as well as associated post-synaptic long-term potentiation (LTP) after two-vessel occlusion (Zheng et al., 2020).

## 40 Hz sensory stimulation as therapeutics in AD patients

Based on the findings of 40 Hz sensory stimulation in various animal models of AD, efforts have been made to test GENUS in human patients. Although these studies are in their early stages and few reports have been published, the results appear efficacious and promising in impeding AD neuropathology. A pilot study was conducted with 18 AD patients (6 mild, 6 moderate, and 6 severe patients), who were subjected to 40 Hz audio stimulation (*via* speakers) twice a week for 6 weeks. The outcome measurements were performed using the St. Louis University Mental Status Test (SLUMS) and an observed emotion rating scale. The results showed that 40 Hz auditory stimulation had a significantly positive impact on mild to moderate AD patients compared to random visual stimulation (Clements-Cortes et al., 2016).

One study by Suk et al. (2020) delivered 40 Hz sensory stimulation (both audio and visual) to healthy individuals and AD patients with concurrent electroencephalogram (EEG) recording, and epilepsy patients with concurrent recording of intracranial EEG (iEEG). The results confirmed that the stimulation induced coordinated 40 Hz entrainment in all subject groups, without any interictal/ictal spikes during or after stimulation. Results from iEEG confirmed that 40 Hz stimulation could also entrain deeper brain regions without any safety issues (Suk et al., 2020). In this light, another study tested 40 Hz stimulation in prodromal AD patients for longer period (He et al., 2021). Patients received 40 Hz flicker for 1 h daily for either 4 weeks or 8 weeks without any remarkable safety concerns or clinical level tolerability. Magnetic resonance imaging (MRI) data and proteomics analysis of cerebral spinal fluid (CSF) showed that prolonged exposure (after 8 weeks) to gamma sensory flicker could increase neural network connectivity as well as alter cytokine and immune factors in the CSF (He et al., 2021).

Together, these studies further suggest the possible clinical efficacy of 40 Hz sensory stimulation in various disease conditions and could be subjected to clinical trials to establish a therapeutic platform. In fact, a recent randomized placebo-controlled clinical trial (Clinical Trial No: NCT04055376) was conducted in (*n* = 15) mild AD patient volunteers who received 1 h of daily 40 Hz sensory stimulation using the previously described device for 4 months (Chan et al., 2021b). The results showed that 40 Hz GENUS device safely and effectively entrained 40 Hz oscillation in both cortical and subcortical regions. The treatment group showed reduced ventricular dilation and hippocampal shrinkage compared to control groups after 3 months of stimulation. In addition, the treatment group showed enhanced functional connectivity in the default mode network (DMN) as well as the medial visual network (MVN), and also improved circadian rhythm, and improved cognitive functioning (i.e., face-name recall test), over the control group (Chan et al., 2021b).

## Challenges ahead

Despite some of the successful studies reviewed above, it is important to note that some groups have also reported failure to replicate the original results. For example, in a very recent study, Soula et al. (2023) used APP/PS1 and 5xFAD animal models and showed that application of both acute (10 min baseline followed by 1 h stimulation before animals were sacrificed) and chronic (1 h stimulation for consecutive 7 days before animals were sacrificed) 40 Hz visual flickering did not entrain deeper structures. The results further showed no obvious changes in Aβ load or microglia morphology in the animals (Soula et al., 2023). Another study reported that optogenetic stimulation of PV + neurons in 5xFAD mice increased Aβ plaques in the basal forebrain rather than decrease the load (Wilson et al., 2020). One possible reason for such discrepancy is that, because of our lack of understanding of the precise neural mechanism behind gamma entrainment, there is a lack of standardized procedure in the current literature to achieve precision (or even customized) stimulation/entrainment. As such, perhaps some parameters are more optimal than others in inducing 40 Hz entrainment. For instance, in a recent study, (Lee et al., 2021) delivered gamma (32 Hz to 50 Hz) flickering light stimulation using organic LEDs attached to eyeglasses in order to test the optimal color, intensity, and frequency of the stimulus in healthy adults by comparing event-related synchronization (ERS) values of entrained gamma waves. While comparing the propagation of gamma waves from the visual cortex to other brain regions, these authors noticed that red light entrained gamma waves most effectively, followed by white light, while green and blue light entrained the least gamma waves. Furthermore, lights with higher luminance intensities (700 and 400 cd/m^2^) delivered stronger gamma waves than those with lower intensities (100 and 10 cd/m^2^) (Lee et al., 2021). Therefore, parameter that are tailored toward optimal visual and auditory entrainment is likely the next milestone in this field.

Apart from stimulation parameters, another possible reason for variability between studies could be participant/patient specificity and response. The accuracy and functionality of 40 Hz sensory stimulation may differ during various stages of AD pathogenesis, as Aβ load varies during the process. While the results from animal studies imply that 40 Hz sensory stimulation might be efficacious during early stages of AD, this may not be true for patients in other stages. Furthermore, it has been shown that not all AD patients suffer the same kind of pathology in gamma oscillation. For example, some studies have reported an overall decrease in gamma band power or coherence across cortices in patients (Ribary et al., 1991; Stam et al., 2002; Başar et al., 2016), while others have reported an overall increase (Osipova et al., 2006; Rossini et al., 2006; van Deursen et al., 2008, 2011; Başar et al., 2017). These results suggest that there are probably multiple types of gamma pathology, and not all of which is likely to respond to 40 Hz sensory entrainment. In the field of EEG, this is known as the “inverse problem”, where gamma frequency recorded over the scalp can come from countless combinations of different dipoles and waveforms, and we would not be able to know which is which. As such, perhaps multiple forms of AD pathology can all give rise to various alterations in gamma oscillation, which possibly contributes to the increasing variability amongst studies, and perhaps further highlights the need for more mechanistic research (e.g., animal models). In this context, so far we know that studies from transgenic AD animal models have confirmed that the application of extraneous 40 Hz gamma can significantly reduce Aβ load and hyper tau-phosphorylation *via* vasodilation and activation of microglia, although these studies did not intend to reveal the mechanistic involvement of gamma in AD pathogenesis (Iaccarino et al., 2016; Adaikkan et al., 2019; Martorell et al., 2019; Chan et al., 2021a). A recent report by Gaubert et al. (2019) on a large cohort of pre-clinical subjects sheds some light on how gamma oscillation contributes during AD pathogenesis in an attempt to identify a causal relationship between Aβ deposition and gamma power. The study revealed an inverted “U-shaped” relationship where an initial increase in Aβ deposition results in an increase in gamma power which, however, decreases as Aβ deposition surpasses a threshold, causing the breakdown of neural circuits and substantiating AD pathophysiology (Gaubert et al., 2019). These results perhaps imply a compensatory mechanism at the early stage of AD pathogenesis (pre-clinical phase) when neurons respond to Aβ deposition by increasing gamma power. In contrast, further chronic increase in Aβ load destabilizes and starts degenerating neural network machinery, leading to a further decrease in gamma power.

Cognitively and behaviorally, studies have also shown that variability in patient’s cognitive state can also significantly modulate the effectiveness of the intervention. For example, we have previously reported that the application of theta or gamma waves *via* tACS methodologies was able to improve short-term memory only in low-performers, with high-performers showing negligible or no improvements (Tseng et al., 2016, 2018; Juan et al., 2017; Sahu and Tseng, 2021). This could be a similar possibility for AD patients, where high-performers might not respond well to neural entrainment, leaving individual variability as a major covariate to be considered in future studies.

## Opportunities ahead

We think one interesting and possibly advantageous feature of sensory stimulation lies in its highly diffusive nature. Previous non-invasive brain stimulation techniques such as transcranial magnetic stimulation (TMS) and transcranial direct current stimulation (tDCS) have to be applied over the skull, and their effects are therefore minimal and limited to the cortical areas. Other techniques, such as deep brain stimulation (DBS), can reach the subcortical areas but has been designed with focality in mind and requires surgical implantation. As such, the lack of focal precision of sensory entrainment (due to propagation) actually sets it apart from other techniques, and in a good way: non-sensory or subcortical areas such as the hippocampus can also receive gamma entrainment from the highly propagative nature of gamma-entrained sensory signals. In this context, it has been reported that sensory entrainment starts from sensory (auditory or visual) cortices and then propagates to other brain areas, including the medial prefrontal cortex (mPFC) and CA1 area of the hippocampus in animal models (Adaikkan et al., 2019; Martorell et al., 2019) as well as to deeper brain regions in humans (Suk et al., 2020). This could be a major advantage of the application of sensory entrainment in diseases such as AD where neurodegeneration occurs globally in the brain that results in sporadic accumulation of amyloid beta (Aβ) plaques and neurofibrillary tangles. Indeed, such applications have been successfully tested in AD patients (Clements-Cortes et al., 2016; Chan et al., 2021b), and AD is currently the most investigated population in the context of 40 Hz sensory stimulation.

One unexplored, but possibly highly promising, population for 40 Hz sensory stimulation could potentially be traumatic brain injury (TBI). TBI is caused by blunt external force to the head, resulting in life-threatening condition that causes grave disabilities and exhibits severe neuronal damage due to hyper-phosphorylated tau-pathology (in chronic traumatic encephalopathy) and Aβ formation in the intra-axonal regions (Blennow et al., 2016). What sets TBI apart from the rest of neurodegenerative diseases, however, is its wide variance of lesion locations across every patient. As such, no TBI patients are truly identical due to the variety of head trauma one can encounter. Despite such variability, EEG recordings in mild-TBI patients revealed a significant decrease in synchronization of the low gamma band frequency (25 Hz−40 Hz) across the scalp when compared to healthy controls (Wang et al., 2017). Similarly, a recent study showed decreased gamma power in rat models of TBI in which case 40 Hz visual entrainment by blue LED light could revive gamma power in the injury side of the brain in order to improve overall prognosis (Yang et al., 2022). Therefore, it is highly likely that the propagative nature of sensory entrainment may be suitable for a lesion-diverse condition like TBI, and it could also be remarkably beneficial in terms of scavenging hyper-phosphorylated tau protein and Aβ clusters, thus having significant prognostic value. To our knowledge no study to date has applied GENUS to TBI patients, though this is worth trying given the results we have reviewed so far with 40 Hz entrainment.

Another potentially interesting, but highly speculative, direction is that perhaps 40 entrainment will not work for other diseases/disorders because each disease/disorder has its own specific frequency. For example, Parkinson’s disease (PD), characterized by resting tremor, bradykinesia, and rigidity, was reported to have abnormal gamma oscillation not at 40 Hz, but from 41 Hz to 45 Hz, which correlated with tremor (Brazhnik et al., 2021). Similarly, frequency range of 60 Hz–90 Hz is involved in bradykinesia (Swann et al., 2016). In the same vein, studies from TBI patients showed relatively low frequency gamma range of 25 Hz–40 Hz compared to healthy subjects (Wang et al., 2017). Or, patients with schizophrenia have shown deficits in high gamma (≥ 60 Hz) that is coupled with visual processing (Grützner et al., 2013). In addition, patients with schizophrenia have also been shown to exhibit low gamma range (24 Hz–48 Hz) occipital evoked activity, where gamma ranges within 40–100 Hz are also associated with spectral abnormality in the disease (Hirano and Uhlhaas, 2021). Therefore, the variations of gamma frequency in various diseases may substantiate the applications of sensory entrainment in a disease-tailored manner. Such application of sensory entrainment in various patients might establish new directions to discover further therapeutic strategies. Though, it is important to note that EEG signals in and of itself does not reflect etiology, but some kind of downstream manifestation of the actual causes. As such, targeting specific abnormal oscillatory frequency with sensory entrainment may successfully entrain one’s EEG but at the same time fail to improve one’s condition (i.e., the inverse inference problem in EEG). Therefore, more animal model studies are needed to draw a complete picture of the neural mechanism(s) underlying sensory entrainment.

## Conclusion

Altogether, 40 Hz sensory stimulation has shown promising effects in AD patients (Clements-Cortes et al., 2016; Chan et al., 2021b; He et al., 2021), and we think the diffusive nature of sensory stimulation makes it an unique tool apart from TMS, tDCS, and DBS, with possible potential for treating TBI. More animal model studies are needed to provide deeper understanding of its underlying mechanism(s), and thus the extent of its translational potential, to different patient populations.

## Author contributions

PS and PT conducted literature review and wrote the manuscript. Both authors contributed to the article and approved the submitted version.
